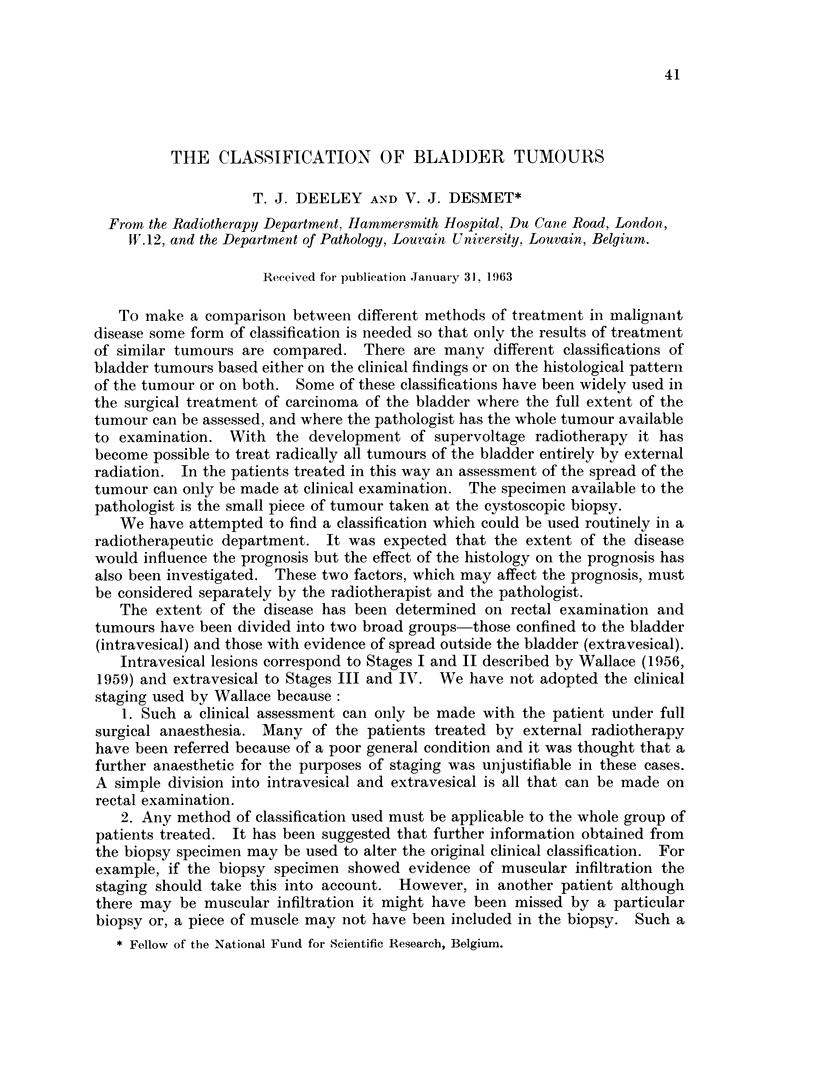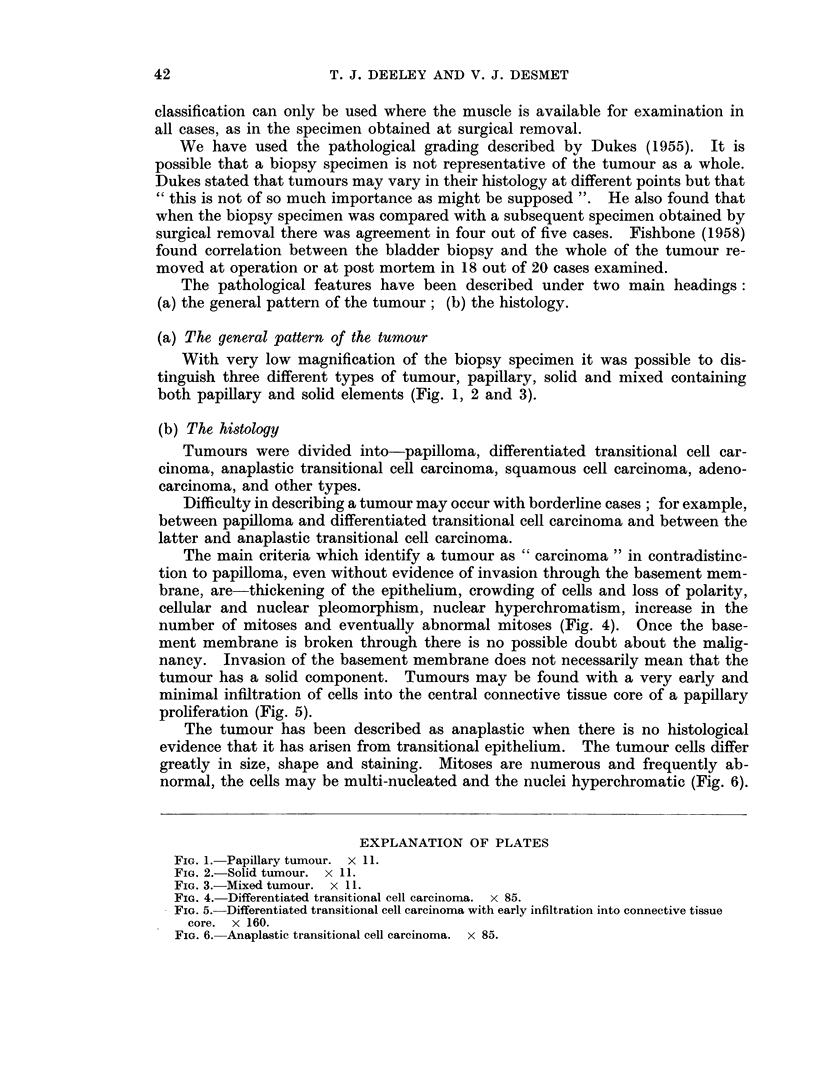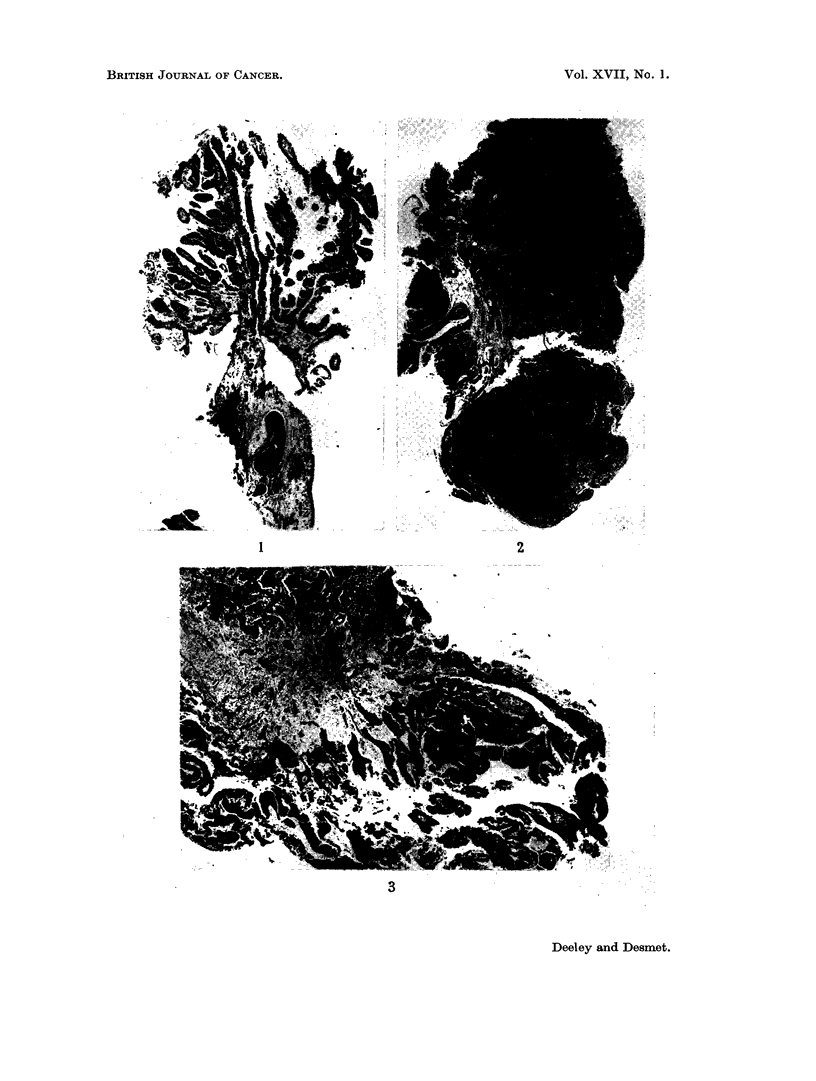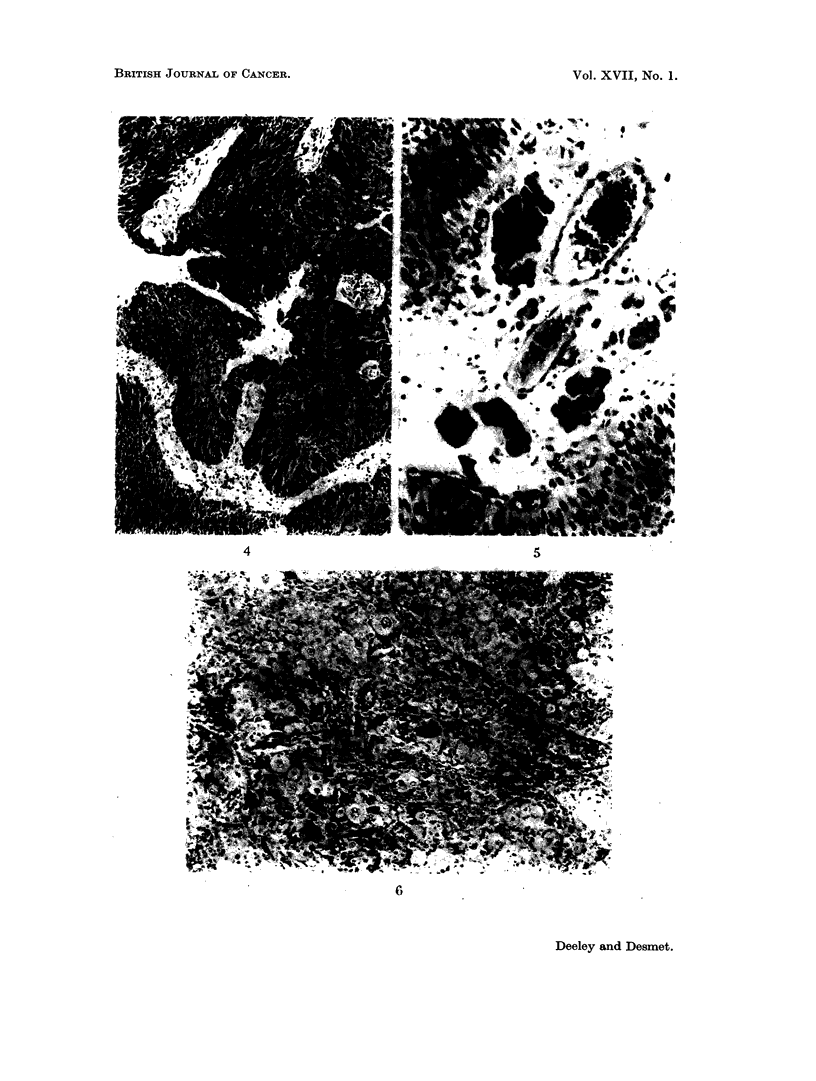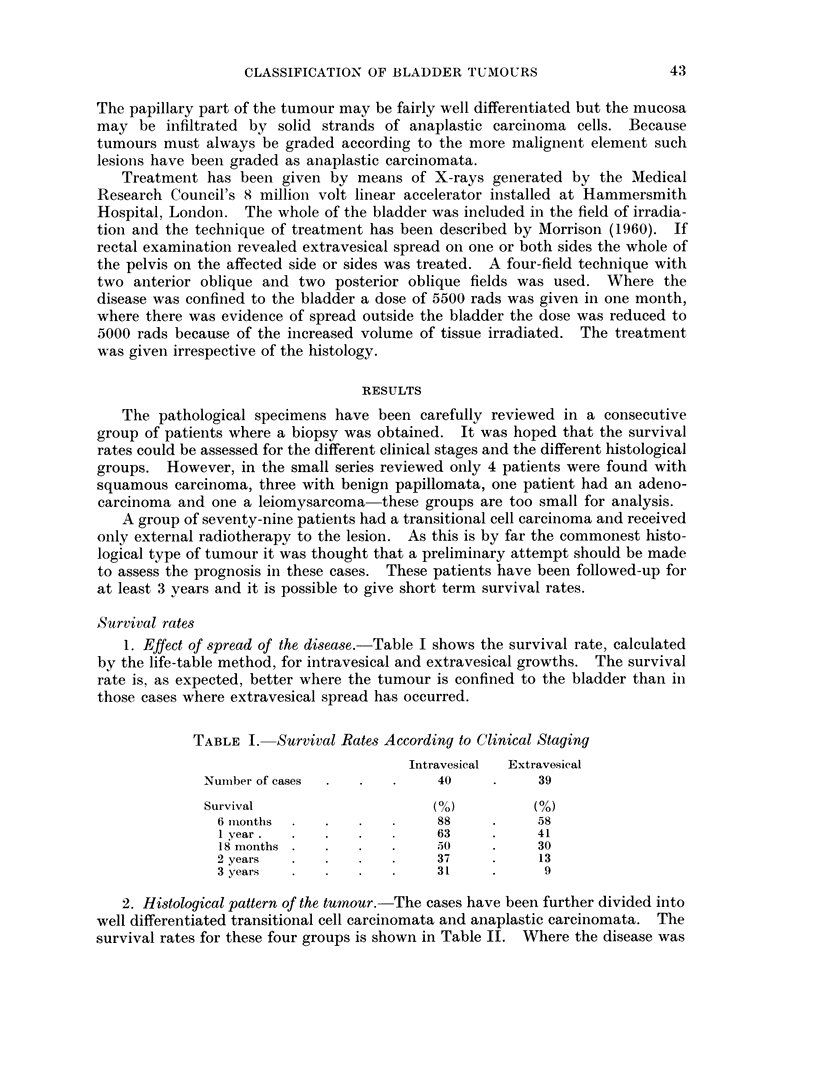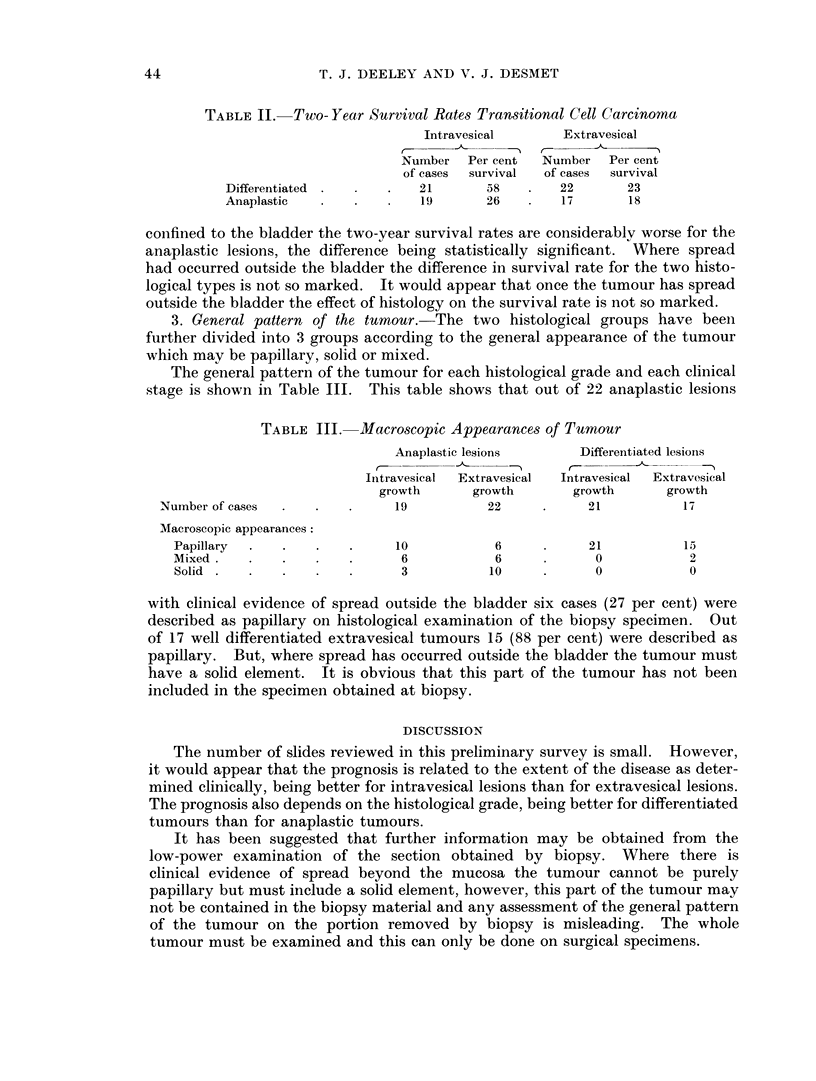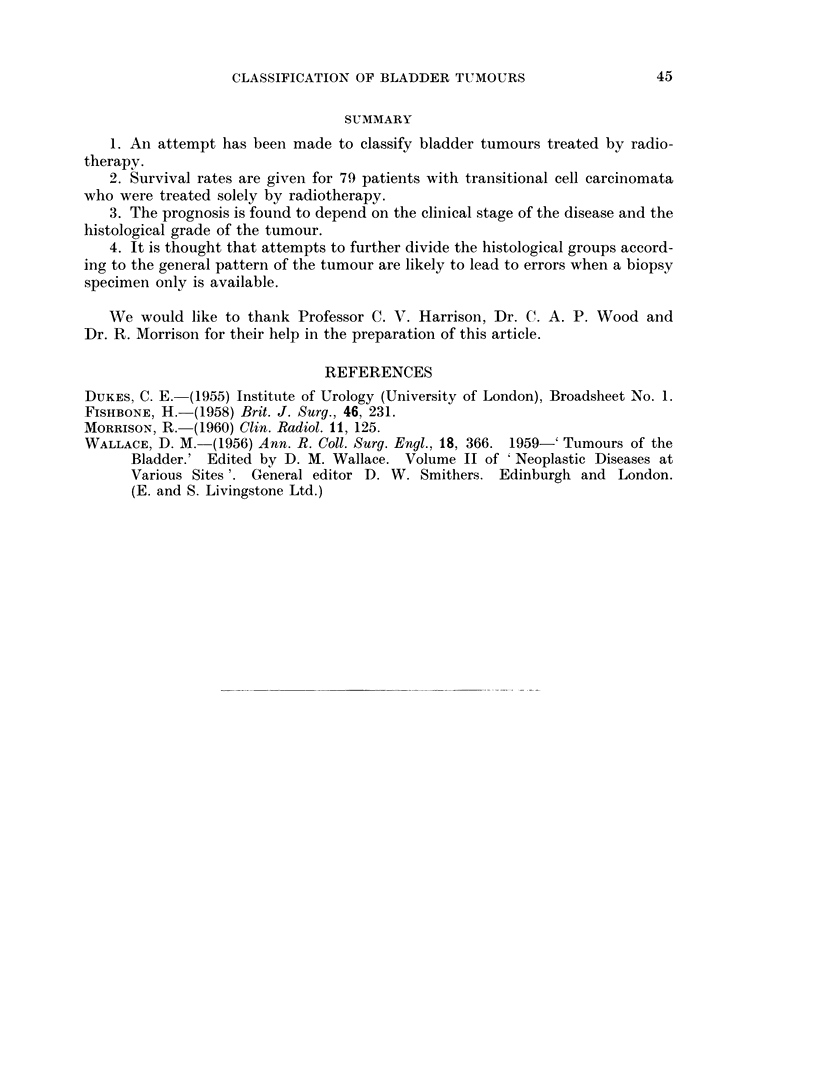# The Classification of Bladder Tumours

**DOI:** 10.1038/bjc.1963.5

**Published:** 1963-03

**Authors:** T. J. Deeley, V. J. Desmet

## Abstract

**Images:**


### THE CLASSIFICATION OF BLADDER TUMOURS

T. J. DEELEY AND V. J. DESMET*

*From the Radiotherapy Department, Hammersmith Hospital, Du Cane Road, London, W.12, and the Department of Pathology, Louvain University, Louvain, Belgium.*

Received for publication January 31, 1963

To make a comparison between different methods of treatment in malignant disease some form of classification is needed so that only the results of treatment of similar tumours are compared. There are many different classifications of bladder tumours based either on the clinical findings or on the histological pattern of the tumour or on both. Some of these classifications have been widely used in the surgical treatment of carcinoma of the bladder where the full extent of the tumour can be assessed, and where the pathologist has the whole tumour available to examination. With the development of supervoltage radiotherapy it has become possible to treat radically all tumours of the bladder entirely by external radiation. In the patients treated in this way an assessment of the spread of the tumour can only be made at clinical examination. The specimen available to the pathologist is the small piece of tumour taken at the cystoscopic biopsy.

We have attempted to find a classification which could be used routinely in a radiotherapeutic department. It was expected that the extent of the disease would influence the prognosis but the effect of the histology on the prognosis has also been investigated. These two factors, which may affect the prognosis, must be considered separately by the radiotherapist and the pathologist.

The extent of the disease has been determined on rectal examination and tumours have been divided into two broad groups—those confined to the bladder (intravesical) and those with evidence of spread outside the bladder (extravesical).

Intravesical lesions correspond to Stages I and II described by Wallace (1956, 1959) and extravesical to Stages III and IV. We have not adopted the clinical staging used by Wallace because :

1. Such a clinical assessment can only be made with the patient under full surgical anaesthesia. Many of the patients treated by external radiotherapy have been referred because of a poor general condition and it was thought that a further anaesthetic for the purposes of staging was unjustifiable in these cases. A simple division into intravesical and extravesical is all that can be made on rectal examination.

2. Any method of classification used must be applicable to the whole group of patients treated. It has been suggested that further information obtained from the biopsy specimen may be used to alter the original clinical classification. For example, if the biopsy specimen showed evidence of muscular infiltration the staging should take this into account. However, in another patient although there may be muscular infiltration it might have been missed by a particular biopsy or, a piece of muscle may not have been included in the biopsy. Such a

* Fellow of the National Fund for Scientific Research, Belgium.

classification can only be used where the muscle is available for examination in all cases, as in the specimen obtained at surgical removal.

We have used the pathological grading described by Dukes (1955). It is possible that a biopsy specimen is not representative of the tumour as a whole. Dukes stated that tumours may vary in their histology at different points but that " this is not of so much importance as might be supposed ". He also found that when the biopsy specimen was compared with a subsequent specimen obtained by surgical removal there was agreement in four out of five cases. Fishbone (1958) found correlation between the bladder biopsy and the whole of the tumour removed at operation or at post mortem in 18 out of 20 cases examined.

The pathological features have been described under two main headings : (a) the general pattern of the tumour ; (b) the histology.

(a) *The general pattern of the tumour*

With very low magnification of the biopsy specimen it was possible to distinguish three different types of tumour, papillary, solid and mixed containing both papillary and solid elements (Fig. 1, 2 and 3).

(b) *The histology*

Tumours were divided into—papilloma, differentiated transitional cell carcinoma, anaplastic transitional cell carcinoma, squamous cell carcinoma, adenocarcinoma, and other types.

Difficulty in describing a tumour may occur with borderline cases ; for example, between papilloma and differentiated transitional cell carcinoma and between the latter and anaplastic transitional cell carcinoma.

The main criteria which identify a tumour as " carcinoma " in contradistinction to papilloma, even without evidence of invasion through the basement membrane, are—thickening of the epithelium, crowding of cells and loss of polarity, cellular and nuclear pleomorphism, nuclear hyperchromatism, increase in the number of mitoses and eventually abnormal mitoses (Fig. 4). Once the basement membrane is broken through there is no possible doubt about the malignancy. Invasion of the basement membrane does not necessarily mean that the tumour has a solid component. Tumours may be found with a very early and minimal infiltration of cells into the central connective tissue core of a papillary proliferation (Fig. 5).

The tumour has been described as anaplastic when there is no histological evidence that it has arisen from transitional epithelium. The tumour cells differ greatly in size, shape and staining. Mitoses are numerous and frequently abnormal, the cells may be multi-nucleated and the nuclei hyperchromatic (Fig. 6).

---

EXPLANATION OF PLATES

FIG. 1.—Papillary tumour. × 11.
FIG. 2.—Solid tumour. × 11.
FIG. 3.—Mixed tumour. × 11.
FIG. 4.—Differentiated transitional cell carcinoma. × 85.
FIG. 5.—Differentiated transitional cell carcinoma with early infiltration into connective tissue core. × 160.
FIG. 6.—Anaplastic transitional cell carcinoma. × 85.

1

2

3

4

5

6

The papillary part of the tumour may be fairly well differentiated but the mucosa may be infiltrated by solid strands of anaplastic carcinoma cells. Because tumours must always be graded according to the more malignent element such lesions have been graded as anaplastic carcinomata.

Treatment has been given by means of X-rays generated by the Medical Research Council's 8 million volt linear accelerator installed at Hammersmith Hospital, London. The whole of the bladder was included in the field of irradiation and the technique of treatment has been described by Morrison (1960). If rectal examination revealed extravesical spread on one or both sides the whole of the pelvis on the affected side or sides was treated. A four-field technique with two anterior oblique and two posterior oblique fields was used. Where the disease was confined to the bladder a dose of 5500 rads was given in one month, where there was evidence of spread outside the bladder the dose was reduced to 5000 rads because of the increased volume of tissue irradiated. The treatment was given irrespective of the histology.

### RESULTS

The pathological specimens have been carefully reviewed in a consecutive group of patients where a biopsy was obtained. It was hoped that the survival rates could be assessed for the different clinical stages and the different histological groups. However, in the small series reviewed only 4 patients were found with squamous carcinoma, three with benign papillomata, one patient had an adenocarcinoma and one a leiomysarcoma—these groups are too small for analysis.

A group of seventy-nine patients had a transitional cell carcinoma and received only external radiotherapy to the lesion. As this is by far the commonest histological type of tumour it was thought that a preliminary attempt should be made to assess the prognosis in these cases. These patients have been followed-up for at least 3 years and it is possible to give short term survival rates.

*Survival rates*

1. *Effect of spread of the disease.*—Table I shows the survival rate, calculated by the life-table method, for intravesical and extravesical growths. The survival rate is, as expected, better where the tumour is confined to the bladder than in those cases where extravesical spread has occurred.

TABLE I.—*Survival Rates According to Clinical Staging*

| | Intravesical | Extravesical |
|---|---|---|
| Number of cases | 40 | 39 |
| Survival | (%) | (%) |
| 6 months | 88 | 58 |
| 1 year | 63 | 41 |
| 18 months | 50 | 30 |
| 2 years | 37 | 13 |
| 3 years | 31 | 9 |

2. *Histological pattern of the tumour.*—The cases have been further divided into well differentiated transitional cell carcinomata and anaplastic carcinomata. The survival rates for these four groups is shown in Table II. Where the disease was

TABLE II.—*Two-Year Survival Rates Transitional Cell Carcinoma*

| | Intravesical | | Extravesical | |
|---|---|---|---|---|
| | Number of cases | Per cent survival | Number of cases | Per cent survival |
| Differentiated | 21 | 58 | 22 | 23 |
| Anaplastic | 19 | 26 | 17 | 18 |

confined to the bladder the two-year survival rates are considerably worse for the anaplastic lesions, the difference being statistically significant. Where spread had occurred outside the bladder the difference in survival rate for the two histological types is not so marked. It would appear that once the tumour has spread outside the bladder the effect of histology on the survival rate is not so marked.

3. *General pattern of the tumour.*—The two histological groups have been further divided into 3 groups according to the general appearance of the tumour which may be papillary, solid or mixed.

The general pattern of the tumour for each histological grade and each clinical stage is shown in Table III. This table shows that out of 22 anaplastic lesions

TABLE III.—*Macroscopic Appearances of Tumour*

| | Anaplastic lesions | | Differentiated lesions | |
|---|---|---|---|---|
| | Intravesical growth | Extravesical growth | Intravesical growth | Extravesical growth |
| Number of cases | 19 | 22 | 21 | 17 |
| Macroscopic appearances : | | | | |
| Papillary | 10 | 6 | 21 | 15 |
| Mixed | 6 | 6 | 0 | 2 |
| Solid | 3 | 10 | 0 | 0 |

with clinical evidence of spread outside the bladder six cases (27 per cent) were described as papillary on histological examination of the biopsy specimen. Out of 17 well differentiated extravesical tumours 15 (88 per cent) were described as papillary. But, where spread has occurred outside the bladder the tumour must have a solid element. It is obvious that this part of the tumour has not been included in the specimen obtained at biopsy.

### DISCUSSION

The number of slides reviewed in this preliminary survey is small. However, it would appear that the prognosis is related to the extent of the disease as determined clinically, being better for intravesical lesions than for extravesical lesions. The prognosis also depends on the histological grade, being better for differentiated tumours than for anaplastic tumours.

It has been suggested that further information may be obtained from the low-power examination of the section obtained by biopsy. Where there is clinical evidence of spread beyond the mucosa the tumour cannot be purely papillary but must include a solid element, however, this part of the tumour may not be contained in the biopsy material and any assessment of the general pattern of the tumour on the portion removed by biopsy is misleading. The whole tumour must be examined and this can only be done on surgical specimens.

SUMMARY

1. An attempt has been made to classify bladder tumours treated by radiotherapy.

2. Survival rates are given for 79 patients with transitional cell carcinomata who were treated solely by radiotherapy.

3. The prognosis is found to depend on the clinical stage of the disease and the histological grade of the tumour.

4. It is thought that attempts to further divide the histological groups according to the general pattern of the tumour are likely to lead to errors when a biopsy specimen only is available.

We would like to thank Professor C. V. Harrison, Dr. C. A. P. Wood and Dr. R. Morrison for their help in the preparation of this article.

### REFERENCES

DUKES, C. E.—(1955) Institute of Urology (University of London), Broadsheet No. 1.
FISHBONE, H.—(1958) *Brit. J. Surg.*, **46**, 231.
MORRISON, R.—(1960) *Clin. Radiol.* **11**, 125.
WALLACE, D. M.—(1956) *Ann. R. Coll. Surg. Engl.*, **18**, 366. 1959—'Tumours of the Bladder.' Edited by D. M. Wallace. Volume II of 'Neoplastic Diseases at Various Sites'. General editor D. W. Smithers. Edinburgh and London. (E. and S. Livingstone Ltd.)